# Comparison of the acute toxicity, analgesic and anti-inflammatory activities and chemical composition changes in Rhizoma anemones Raddeanae caused by vinegar processing

**DOI:** 10.1186/s12906-019-2785-0

**Published:** 2020-01-15

**Authors:** Sha-Sha Wang, Shao-Yan Zhou, Xiao-Yan Xie, Ling Zhao, Yao Fu, Guang-Zhi Cai, Ji-Yu Gong

**Affiliations:** Changchun University of Chinese Medicine, Boshuo Road No. 1035, Jingyue High-tech Industrial Development Zone, Changchun, 130117 China

**Keywords:** *Rhizoma anemones Raddeanae*, Vinegar processing, Anti-inflammation, Analgesia

## Abstract

**Background:**

As the dry rhizome of *Anemone raddeana* Regel, *Rhizoma Anemones Raddeanae* (RAR), which belongs to Ranunculaceae, is usually used to treat wind and cold symptoms, hand-foot disease and spasms, joint pain and ulcer pain in China. It is well known that the efficacy of RAR can be distinctly enhanced by processing with vinegar due to the reduced toxicity and side effects. However, the entry of vinegar into liver channels can cause a series of problems. In this paper, the differences in the acute toxicity, anti-inflammatory and analgesic effects between RAR and vinegar-processed RAR were compared in detail. The changes in the chemical compositions between RAR and vinegar-processed RAR were investigated, and the mechanism of vinegar processing was also explored.

**Methods:**

Acute toxicity experiments were used to examine the toxicity of vinegar-processed RAR. A series of studies, such as the writhing reaction, ear swelling experiment, complete Freund’s adjuvant-induced rat foot swelling experiment and cotton granuloma, in experimental mice was conducted to observe the anti-inflammatory effect of vinegar-processed RAR. The inflammatory cytokines of model rats were determined by enzyme-linked immunosorbent assay (ELISA). Liquid Chromatography-Quadrupole-Time of Flight mass spectrometer Detector (LC-Q-TOF) was used to analyse the chemical compositions of the RARs before and after vinegar processing.

**Results:**

Neither obvious changes in mice nor death phenomena were observed as the amount of vinegar-processed RAR in crude drug was set at 2.1 g/kg. Vinegar-processed RAR could significantly prolong the latency, reduce the writhing reaction time to reduce the severity of ear swelling and foot swelling, and remarkably inhibit the secretion of Interleukin-1β(IL-1β), Interleukin-6 (IL-6) and tumor necrosis factor-α (TNF-α) proinflammatory cytokines. The content of twelve saponins (e.g., *Eleutheroside K*) in RAR was decreased after vinegar processing, but six other types (e.g., RDA) were increased.

**Conclusions:**

These results revealed that vinegar processing could not only improve the analgesic and anti-inflammatory effects of RAR but also reduce its own toxicity.

**Trial registration:**

Not applicable.

## Background

*Rhizoma Anemones Raddeanae* (RAR) is the dry rhizome of *Anemone raddeana* Regel, which belongs to Ranunculaceae. It is used to treat rheumatism and has been applied clinically in various fields for conditions such as wind and cold symptoms, hand-foot disease and spasms, joint pain and ulcer pain in China. Zheng’s study [1] showed that RAR has an obvious anti-inflammatory effect on the classical inflammatory reaction. The extract of RAR can reduce the ear swelling of mice caused by xylene and foot swelling of rats caused by fresh egg white. Additionally, the RAR extract also decreases the proliferation of cotton ball granulosis in experimental rats. Zhang et al. [2] reported that the ethanolic extract of RAR could improve the primary and secondary inflammation of rats with adjuvant arthritis, and this improvement might be achieved by reducing the inflammatory factors of IL-1 beta, IL-6, IL-10 and TNF- alpha. In previous work, Gong et al. [3] indicated that the active groups of *BU-6E* in RAR had significant anti-inflammatory effects on RAW 264.7 cells. The anti-inflammatory effect occurred in both a time- and dose-dependent manner.

Vinegar-processed RAR, which has been performed according to Beijing Chinese medicine processing standards [4], has been used in the traditional ancient prescription of Zaizao pills. It was reported that processing with vinegar can reduce the toxicity or side effects of *Radix Bupleuri* to enhance its drug efficacy [5]. However, the entry of vinegar into liver channels can cause a series of effects, such as astriction, detoxification, water-following process, and analgesia.

Compared with the published researches which preliminarily/detailedly demonstrate the pharmacology effect of RAR [1–3], no related report has been published to discuss neither the pharmacology action of the vinegar-processed RAR nor the composition change of RAR in the vinegar-processing. In this paper, the comparison of differences in acute toxicity [6, 7], anti-inflammatory and analgesic effects between RAR and vinegar-processed RAR was conducted systematically. The changes in the chemical composition of RAR and vinegar-processed RAR were investigated, and the mechanism of vinegar processing was explored preliminarily to provide available scientific support for the mechanism study and clinical application of vinegar-processed RAR [8, 9].

## Methods

### Materials

#### Reagents

RAR medicine was purchased from Xiancao Herb Medical Development Co. Ltd. (batch number: 170522; Jilin, China). The RAR was identified as the dry rhizome of *Anemone raddeana* Regel, which belongs to Ranunculaceae and was classified according to Chinese Pharmacopeia (2015).

The *Raddeanin A* (RDA) reference substance (purity ≥98%) was obtained from the National Institutes for Food and Drug Control (batch number: 89412793; Beijing, China). Rice vinegar was purchased from Beijing Er Shang Longhe Food Co., Ltd, (Beijing, China). Acetic acid and methanol were of chromatographic grade, and other reagents were of analytical grade. Ultra-pure water was applied throughout the experiment. Xylene was purchased from Beijing Chemical Reagent Factory (batch number: 20170216; Beijing, China). Glacial acetic acid was purchased from Beijing Chemical Reagent Factory (batch number: 20160818; Beijing, China). Sodium carboxymethyl cellulose (CMC-Na; 0.1%) was purchased from Beijing Chemical Reagent Factory (batch number: 20170120; Beijing, China). Complete Freund’s adjuvant was obtained from Beijing Ding Guo Chang Sheng Biotechnology (batch number: AC-0051; Beijing, China). IL-1β, IL-6 and TNF-α ELISA kits were manufactured by R&D Systems inc. (batch numbers: IL-1β (RLB00), IL-6 (R6000B), and TNF-α ELISA (RTA00); USA).

#### Animals

Kunming mice (SPF level) weighing 18 to 22 g and SD rats weighing 220 to 240 g were all provided by Yi Si Experimental Animal Centre (animal certificate number: SCXK (JI)-2016–0002; Jilin, China). Both mice and rats were healthy and were never used for experiments. All the experiments were conducted according to the guidelines of the National Research Committee and Animal Ethics Committee of Changchun University of Traditional Chinese Medicine. The rats and mice were placed in a standard breeding room. Every ten mice and five rats were placed into one cage respectively to be separated from each other. The ambient temperature was 18–22 °C. The temperature was recorded daily and was adjusted if necessary. The wet and dry bulb thermometers were used to estimate and measure the humidity every day. The relative humidity was adjusted within the range of 50–60%. The noise was set below 60 dB. The lights were turned on at 8:00 and turned off at 20:00 every day. The ventilation was operated 8 to 20 times per hour with an air flow rate of 10 to 25 cm^3^ per minute. The litter in the cages of mice and rats was cleaned every 2 days. The cages were washed and disinfected once a week. Clean, pollution-free drinking water was supplied timely with drinking bottles, which were washed every 2 days. The breeding room was sprayed with 0.1% benzalkonium perm spray every month and was sterilized with peracetic acid every quarter.

#### Instruments

HPLC analysis was carried out using a Shimadzu Model 2030 HPLC instrument (Shimadzu, Japan). Drugs were weighed using an AB135-S electronic analytical balance (Mettler-Toledo, Shanghai, China). Ultrasonic treatment was carried out using a KQ3200 DB CNC ultrasonic cleaning device (KunShan Ultrasonic Instruments Co., Ltd., Shanghai, China). An HH-S24 electric constant temperature water bath (Jintan Automation Instruments Co., Ltd., Jiangsu, China) was used for heating. The intelligent hot plate YLS-6B (Shanghai Precision Instrument Co., Ltd., Shanghai, China) was used in mouse hot-plate experiments. A Vernier caliper (Measuring Instruments Co., Ltd. Nanjing Su) with a scale range of 0–150 nm was used in the complete Freund’s adjuvant-induced rat foot-swelling experiments. The optical density (OD) values were measured using an iMark microplate reader (Bio-Rad, USA). The mass spectrum results were obtained using an LC-Q-TOF mass spectrometer (Mass Hunter workstation; Agilent, USA).

### Pharmacological experimental study

#### Preparation of samples for experiment

RAR sample: 210 g of crude RAR was ground, followed by extraction with water three times. Thereafter, decoction was conducted with 8 volumes of water for 2 h, followed by 6 volumes of water for 1.5 h, and then 6 volumes of water for 1 h. The obtained extract solution was collected and evaporated to dryness. The extraction yield of RAR was calculated as 16.84%.

Vinegar-processed RAR sample: 210 g of crude RAR was mixed with 42 mL of vinegar at the ratio of 5:1. After soaking for 2 h, the mixture was stir-fried at 120 °C for 10 min. The extraction steps were the same as that of RAR, and the extraction yield of vinegar-processed RAR was calculated as 16.54%.

According to the Chinese Pharmacopoeia (2015), the dosage of RAR was in the range of 1 to 3 g, and the middle value of 2 g was adopted in this study. Thus, the dosage of 2 g was selected to strengthen the pharmacological effects and select the effective component in RAR. According to the average weight of people at 60 kg, the normal dosage of crude drug was set as 0.03 g/kg of body weight. Thus, the dose of crude drugs of 5 times, 10 times, and 20 times, corresponding to 1.05 g/kg, 2.1 g/kg, and 4.2 g/kg, respectively, were chosen as the low, medium and high doses, respectively. Considering the extraction yield of 16, 0.1% CMC-Na was used to prepare three solutions at different concentrations (168 mg/kg, 336 mg/kg and 672 mg/kg) before oral administration for the initial efficacy experiment.

#### Acute toxicity experiment in mice

The fifty mice fasted for 12 h before experiment were randomly divided into five equal groups as follows: blank group, RAR group 1, RAR group 2, vinegar-processed RAR group 1, and vinegar-processed RAR group 2. Each group consisted of ten mice (male and female in half). RAR group 1 and vinegar-processed RAR group 1 were intragastrically administered once a day with 4.2 g/kg of crude drug based on the weight of each mouse (ig). RAR group 2 and vinegar-processed RAR group 2 were intragastrically administered once a day with 2.1 g/kg of crude drug based on the weight of each mouse (ig). The blank group was intragastrically administered with an equal volume of distilled water. The mice were observed per 15-min interval since being intragastrically administered from 0 to 2 h, per 0.5-h interval from 2 to 4 h, per 1-h interval from 4 to 8 h, and per 4-h interval from 8 to 24 h, respectively. Daily observation was performed for 14 days. The weight and intake conditions of the mice were recorded, as well as the potential toxic and death phenomena [10–13].

#### Writhing reaction induced by acetic acid in mice

The forty mice (20 male and 20 female) were divided into five groups. The positive control group was given 3 mg/kg of indomethacin, and the blank group was intragastrically administered with 10 mL/kg of distilled water. RAR and vinegar-processed RAR at 2.1 g/kg was intragastrically administered once a day for 7 days. The mice were then injected intraperitoneally (ip) with 10 mL/kg of 0.6% acetic acid 1 h after the last administration, and the time of the first writhing reaction that occurred after three minutes of incubation was recorded as the latency. The writhing reaction times for each mouse within 15 min after modelling was also recorded [14, 15].

#### Hot-plate study in mice

The time since the mice started to lick the hind feet was viewed as the pain threshold. The forty female mice (referring to the pharmacological experimental method [16], hot plates would burn the genitals of male mice) with a pain threshold between 5 s and 30 s were selected and divided randomly into four groups and were intragastrically administered as mentioned above. After 6 days of continuous intragastric administration, the values of the pain threshold after 0.5-h, 1.5-h and 2-h administration of each group were determined by the hot-plate method [17, 18].

#### Xylene induces ear swelling in mice

The twenty male mice and twenty female mice were randomly and equally divided into the model group, positive control group, RAR group and vinegar-processed RAR group. The positive control group was intragastrically administered 100 mg/kg of indomethacin. Additionally, 10 mL/kg of distilled water was intragastrically administered to the blank group. RAR and vinegar-processed RAR were intragastrically administered with 2.1 g/kg once a day for 7 days. After 1 h of the last intragastric administration, all the right auricles of the mice were evenly coated with 40 μL of xylene, and the mice were sacrificed by cervical dislocation after 30 min of xylene treatment. The aures unitas were carefully removed and punched at the same place with the same pore diameter. The aures unitas were weighed immediately to calculate ear swelling [19, 20]. The equation was as follows:$ES\left(\%\right)=\frac{\left( REW- LEW\right)}{LEW}\times 100\%$

ES was utilized to explain ear swelling. REW was utilized to explain the weight of the right ear. LEW was utilized to explain the weight of the left ear.

#### Complete Freund’s adjuvant-induced rat foot swelling

The forty male rats (referring to the pharmacological experimental method, with recommendations for male rats) were randomly and equally divided into the blank group, positive control group, RAR group and vinegar-processed RAR group. The rats in the blank group were intragastrically administered 10 mL/kg of distilled water. Next, 5 mg/kg of methotrexate was intragastrically administered to the positive control group. The RAR and vinegar-processed RAR were intragastrically administered with 2.1 g/kg of RAR and vinegar-processed RAR once a day for 6 days (according to previous experiments, the optimal dosing period was 6 days; thus, 6 days was chosen). After 1 h of the last administration, 100 μL of Freund’s adjuvant was injected subcutaneously into the right hind foot of the rats. The volume of the right hind foot was measured at 0.5 h, 1 h, 2 h, 3 h, 4 h and 6 h after modelling. The foot swelling was defined as the difference in the value of the right posterior foot volume before and after administration [21–23]. The inhibition rate of the foot swelling degree was calculated using the following equation:

$$ TR\left(\%\right)=\frac{\left( BAD- RAD\right)}{BAD}\times 100\% $$

TR was utilized to explain the tumescent inhibition rate. BAD was utilized to explain the average degree of swelling in the blank group. RAD was utilized to explain the average swelling degree of the RAR group or vinegar-processed RAR group.

#### Granuloma model

The fifty male mice (referring to Xu Shuyun’s third edition of pharmacology experimental methodology, with recommendations for male mice) were implanted with approximately 10 mg of sterilized cotton balls by axillary subcutaneous surgery under sterile conditions. Penicillin was dropped onto the wound twice a day postoperatively. The mice were randomly divided into five groups—the blank group, model group, positive control group, RAR group, and vinegar-processed RAR group. Intragastric administration was conducted once a day, and then the mice were sacrificed at the seventh day after continuous administration. The wet weight of the cotton balls removing the granulation tissue was determined. After drying at 60 °C in an oven for three days, the dry weight of the cotton balls was determined to calculate the granuloma weight [24, 25].

#### Determination of inflammatory factors

The blood samples of rats that have been under complete Freund’s adjuvant-induced foot swelling were taken from their abdominal aorta. The obtained samples were then separated, followed by extraction of the serum and plasma by centrifugation. Next, the rats were sacrificed by cervical dislocation. The inflammatory factors IL-1β, IL-6 and TNF-α were measured strictly according to the specification of the ELISA Kit. The OD values were measured using the iMark reader at 450 nm.

#### Statistical methods

All the data were expressed as the mean values ± standard deviation (SD). The data were subjected to statistical analysis of variance (ANOVA) by comparing means with Tukey’s experiment (*p* < 0.05). The IBM SPSS 20 statistical programme was used for all statistical analyses.

### Chemical composition analysis

#### Preparation of reference substance

The moderate RDA reference was weighed accurately and dissolved in a volumetric flask of 5 mL with methanol. The concentration of the solution was fixed at 1.07 mg/mL [26].

#### Preparation of the experiment sample

RAR powder (2.5 g) was screened with a No. 5 sieve and dissolved with 70% ethanol in a 50-mL conical flask as described previously. According to previous studies, 70% ethanol has the highest efficiency to extract substances from drugs. After 40 min of ultrasonic treatment, the solution was filtered, and the filtrate was evaporated to dryness. The residue was dissolved and diluted with methanol to 25 mL.

#### Liquid chromatography (LC) and mass spectrometry (MS) conditions

LC conditions: The ZORBAX SB-C18 column (50 mm × 2.1 mm, 5 μm) was used as the chromatographic column. Gradient elution was carried out with acetonitrile-0.1% formic acid aqueous solution. The flow rate was set as 0.3 mL/min. The column temperature was fixed at 30 °C, and the injection volume was 1 μL.

MS conditions: ESI was selected as the ion source, and MS1 was chosen as the collection method. The acquisition range varied from 100 to 2000. The dry gas temperature was set as 35 °C, and the flow rate was fixed at 8 L/min. The spray pressure was set as 35 psi, and negative ion was selected as the ion type [27–30].

## Results

### Pharmacological results

#### Acute toxicity in mice

**Fig. 1:**
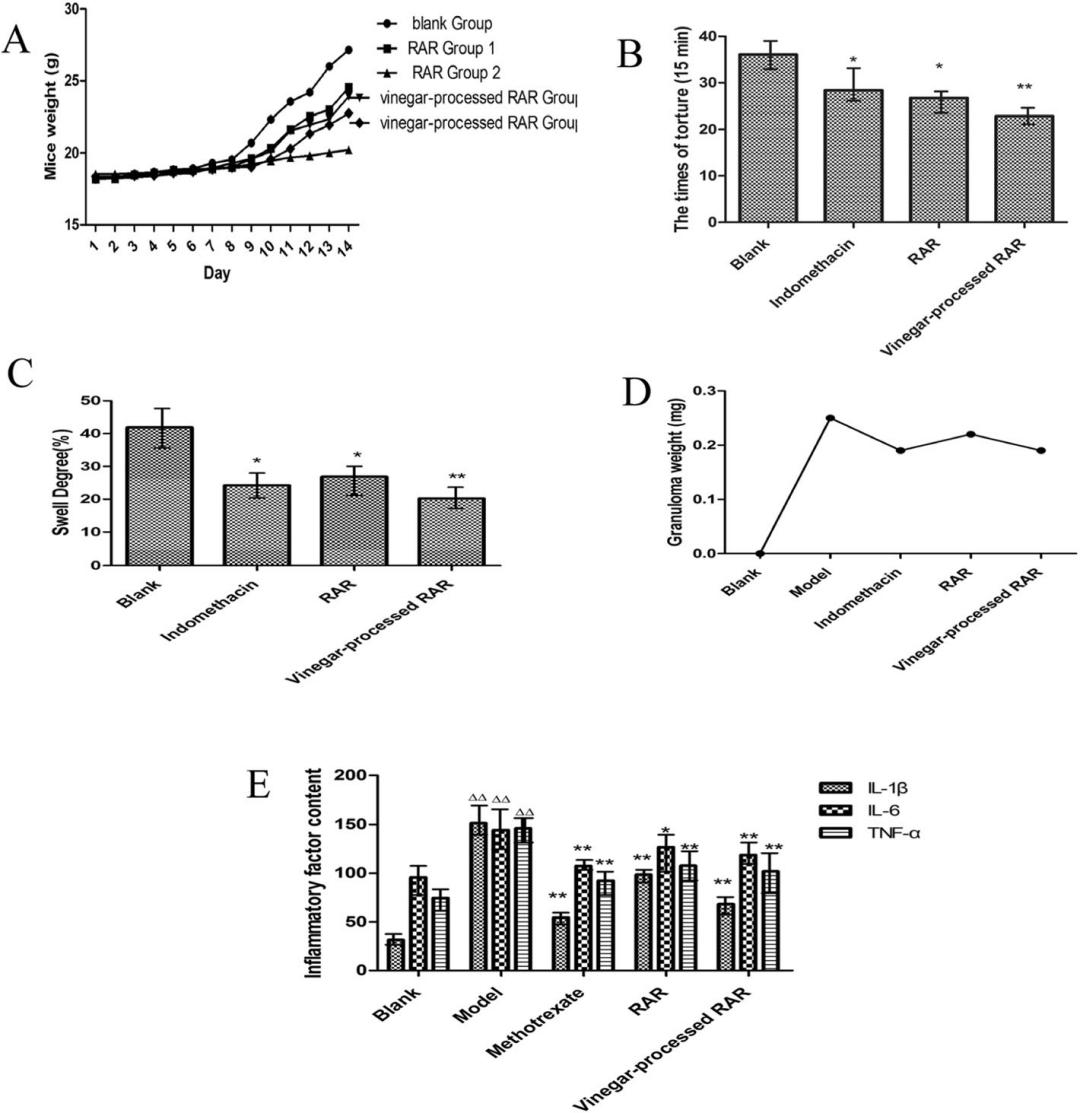
A The weight change map of mouse in acute toxic experiment (Note: RAR group 1 was intragastrically administered once a day with 4.2 g/kg of crude drug based on the weight of each mouse (ig),and vinegar-processed RAR group 1was intragastrically administered once a day with 4.2 g/kg of vinegar-processed crude drug based on the weight of each mouse (ig).RAR group 2 was intragastrically administered once a day with 2.1 g/kg of crude drug based on the weight of each mouse (ig),and vinegar-processed RAR group 2 intragastrically administered once a day with 2.1 g/kg of vinegar-processed crude drug based on the weight of each mouse (ig). *n* = 10). B Effect of RAR and its processed products on the writhing reaction in mice (Note: compared with the blank Group *P < 0.05, **P < 0.01, n = 10). C Effects of RAR and processed products on ear swelling in mice (Note: compared with the model control Group *P < 0.05, **P < 0.01, *n* = 10). D Cotton ball weight in rats (Note: compared with the model Group *P < 0.05, **P < 0.01, *n* = 10). E Inflammatory factor contents of IL-1β, IL-6 and TNF-α levels in each group (Note: compared with the blank Group: *P < 0. 05, **P < 0. 01, compared with the model Group: ^Δ^*P* < 0.05, ^ΔΔ^*P* < 0.01, *n* = 10). See Additional file 1 for additional data.

After intragastric administration with a certain volume of RAR and vinegar-processed RAR, one mouse was found dead and one was in poor condition in RAR group 1. No dead mice were found in RAR group 2. Regarding the vinegar-processed RAR groups, the number of dead mice was one and zero for groups 1 and 2, respectively. Neither visible changes nor death of mice nor obvious differences in the physiological activity occurred between the RAR groups and vinegar-processed RAR groups in the middle dose groups compared with those of the blank group after 24 h of observation. It was suggested that no acute toxicity occurred with the middle dose of RAR or vinegar-processed RAR for the mice. Based on these results, the middle doses of RAR and vinegar-processed RAR were used for subsequent experiments. After the mouse acute poisoning minimum lethal dose (MLD) test, the median lethal dose (LD50) value of the vinegar-processed RAR was 112.64 g/kg, and the MLD value was temporarily 151.14 g of crude drug/kg·d. The weight changes of the mice are summarized and shown in Fig. 1a and Additional file 1 below.

**Fig. 2:**
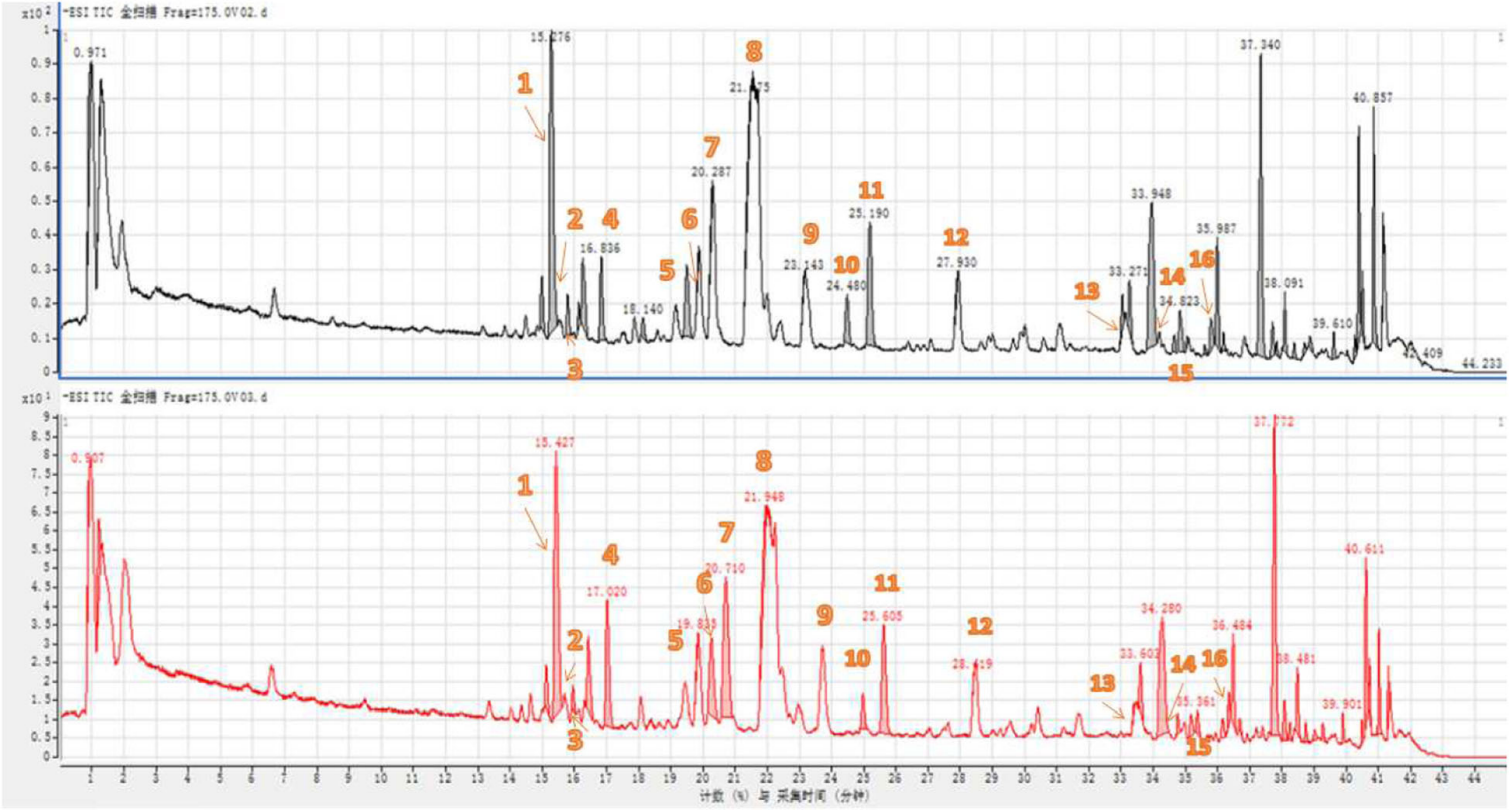
Contrasting total ion chromatogram of RAR and vinegar-processed RAR (See Additional file 1 for additional data)

#### Effect of the writhing reaction induced by vinegar-processed RAR in mice

As shown in Fig. 1b and Additional file 1, compared with the blank group, the use of vinegar-processed RAR significantly extended the latency of the writhing reaction as well as reduced the torture times in mice effectively.

#### Effect of the pain threshold in mice subjected to a hot plate

According to the observation of experimental phenomena, the data of pain thresholds fluctuated and some were repeated after 2.0 h of administration. It was deduced that the memory of mice caused by the chaos of the pain threshold might be formed after repeated stimulation. However, based on the consideration mentioned above, although there was a remarkable increase in the pain threshold in the vinegar-processed RAR group and positive control group after 1.0 h of administration, the data were neglected in this work.

#### Effect of ear swelling induced by vinegar-processed RAR in mice

As shown in Fig. 1c and Additional file 1, RAR could obviously reduce the swelling degree of the ears of mice with significant differences (*P* < 0.05) compared with that of the model group. A more prominent phenomenon of swelling degree could be observed in the vinegar-processed RAR group with a more significant difference (*P* < 0.01).

#### Effect on foot swelling induced by vinegar-processed RAR in rats

In Table 1, the degree of foot swelling of the model group increased to 80.32% compared with that of the blank group, indicating that the model was set up successfully. After 4 h, the foot swelling degree of the foot was significantly decreased in each drug group. Compared with the model group, the foot swelling degree of the vinegar-processed RAR group was decreased, which was significantly different from that of the model group at 2 h. The foot swelling degree of vinegar-processed RAR was much higher than that of the RAR Group, indicated that the vinegar-processed RAR is more effective in the foot swelling treatment.

**Table 1:** Effects of the swelling of feet in rats induced by vinegar-processed RAR ($\overline{\mathrm{x}}$ *± s*, *n* = 10)

Group	Blank group	Model group	Methotrexate group	RAR group	Vinegar-processed RAR group
Dose (mg/kg)	0	0	5	336	336
0.5 h Swell (mm)	4.32 ± 0.50	7.79 ± 0.84	6.83 ± 0.95	7.22 ± 0.45	7.13 ± 0.55
0.5 h Inhibition Rate (%)	0%	80.32%	58.10%	67.13%	65.05%
1.0 h Swell (mm)	4.92 ± 0. 83	8.42 ± 0.56	7.45 ± 0.42	7.41 ± 0.99	7.02 ± 0.23
1.0 h Inhibition Rate (%)	0%	71.13%	51.42%	50.61%	42.68%
2.0 h Swell (mm)	5.21 ± 0. 46	8.57 ± 0.28	7.56 ± 0.25	6.87 ± 0.36^*^	6.56 ± 0.41^*^
2.0 h Inhibition Rate (%)	0%	64.49%	45.11%	31.86%	25.91%
3.0 h Swell (mm)	5.13 ± 0.92	8.32 ± 0.39	6.59 ± 0.68^*^	6.32 ± 0.26^**^	6.28 ± 0.37^**^
3.0 h Inhibition Rate (%)	0%	62.18%	28.46%	23.30%	22.42%
4.0 h Swell (mm)	4.97 ± 0. 71	7.95 ± 0.17	6.33 ± 0.48^**^	6.08 ± 0.78^**^	6.02 ± 0.21^**^
4.0 h Inhibition Rate (%)	0%	60.00%	27.36%	22.33%	21.13%
6.0 h Swell (mm)	4.33 ± 0. 19	7.21 ± 0.44	5.02 ± 0.74^**^	5.30 ± 0.59^**^	4.98 ± 0.29^**^
6.0 h Inhibition Rate (%)	0%	66.51%	15.94%	22.40%	15.01%

#### Effect on the granuloma model reaction induced by vinegar-processed RAR in rats

In Fig. 1d and Additional file 1, compared with the blank group, the cotton ball weight of the vinegar-processed RAR group was reduced, displaying a significant difference. However, the difference in the cotton ball wet weight between the vinegar-progressed RAR group and positive control group was negligible, indicating that the vinegar-processed RAR could be utilized as an effective candidate to inhibit the inflammation proliferative phase.

### Determination of inflammatory factors in inflammatory rats

Standard curves of IL-1β, IL-6 and TNF-α were established according to the specification of the ELISA Kit. Inflammatory factor contents were calculated and are summarized in Fig. 1e and Additional file 1.

Compared with the blank group, the contents of inflammatory factors were increased by 120 pg/mL, 49 pg/mL, and 72 pg/mL, corresponding to nearly 5 times, 1.5 times and 2 times, respectively. Compared with the model group, the contents of the inflammatory factors of IL-1β, IL-6 and TNF-α were significantly decreased in the vinegar-processed RAR group, which effectively reduced the inflammatory cytokine content and achieved the anti-inflammatory effect.

### Mass spectrum results

The chemical structures of the major saponins in RAR were analysed by MS analysis [30, 31].

These saponins in RAR would interact with each other due to vinegar processing. The content of some saponins would increase, while others would decrease. As shown in Fig. 2, based on the information obtained from the total ion flow chart and mass spectrum, it can be calculated that the molecular weights of the components in RAR were 734, 750, 896, 912, 1204, 1220, 1236, 1336, 1366, 1382, 1498, and 1528. Due to only the first-order mass spectrometry being obtained, these components may be deduced as *Eleutheroside K, Raddeanoside B, Saponin P E, Raddeanoside R12, Raddeanoside A, Raddeanoside R6, Raddeanoside R13, Raddeanoside D, Hederasaponin B, Raddeanoside R14, Leonloside D, Raddeanoside R15, Raddeanoside R8, Raddeanoside R9, R18, Hederacholichiside F, Raddeanoside R16,* and *Raddeanoside R17, R10*.

Based on the retention time, it can also be concluded that the contents of *Eleutheroside K, Raddeanoside B, Saponin P E, Raddeanoside R12, Raddeanoside R13, Raddeanoside D, Hederasaponin B, Raddeanoside R14, Raddeanoside R15, Raddeanoside R9, R18,* and *Hederacholichiside F* were decreased after RAR vinegar processing. By contrast, the contents of *Raddeanoside A*, *Raddeanoside R6, Leonloside D, Raddeanoside R16, and Raddeanoside R17, R10* were increased.

## Discussion

In recent studies, certain kinds of triterpenoid saponins which separated from RAR have been reported to be responsible for its bioactivities, such as analgesic and anti-inflammation effects [31]. *Raddeanoside A*, for example, has been identified as a classical triterpenoid saponin amongst these components with obvious analgesic and anti-inflammatory effects [16]. It can be deduced that the content of these components may influence the pharmacological effect of RAR. Vinegars, as traditional fermented foods, is not only used to provide health and therapeutic effects due to their bioactive components [32], but also utilized in traditional Chinese PaoZhi processing technique to enhance the pharmacological effect of medicinal materials. Li reported that the hepatoprotective effect of Radix Bupleuri was successfully strengthened after vinegar process, which changed the distribution of contents of the Radix Bupleuri’s saponin components [33]. In this study, acute-subacute inflammatory models under non-specific inflammatory models (mouse ear swelling model and rat foot-swelling model) and the inflammatory proliferation phase (granuloma formation) were selected as animal models to perform more comprehensive anti-inflammatory exploration of vinegar-processed RAR [34, 35]. According to the results obtained in the determination of inflammatory factors in inflammatory rat experiments, it was suggested that vinegar-processed RAR significantly inhibited the expression of proinflammatory cytokines IL-1β, IL-6 and TNF-α. We deduced that vinegar processing can promote the elimination of inflammation by inhibiting the secretion of proinflammatory cytokines and provides the preliminary basis for the study of RAR anti-inflammatory basic research. The results of mouse ear swelling experiments and rat foot-swelling experiments showed that the anti-inflammatory effect of vinegar-processed RAR was much better than that of RAR. Vinegar processing could strengthen the anti-inflammatory effects of the original medicine. The analgesic experiments were carried out by chemical stimulation (writhing reaction induced by glacial acetic acid) and thermal stimulation experiments (hot plate experiment). The analgesic effects, including prolonging the latency of the writhing reaction and increase in the pain thresholds, were caused by vinegar-processed RAR. After vinegar processing, the response of the stimulated mice towards the outside was very slow, indicating that the vinegar processing can improve the effect of analgesia. Based on the results obtained from acute toxicity experiments, we deduced that RAR toxicity was reduced by vinegar processing.

The data in mass spectrometry analysis indicated that the content of some saponins increased, while others decreased after vinegar processing. The cause might be the interaction among the saponins in RAR utilized in the theory of Traditional Chinese medicine as the drug’s tendency and nature, which would change the substance content and achieve the best drug effect. The triterpenoid saponins in the genus Anemone were mainly divided into C-3 monodesmosidic saponins and C-3, 28 bisdesmosidic saponins based on their carbohydrate chain structure. Regarding the monodesmosidic saponins, the fragment ions at C-3 were removed in sequence. Concerning the bisdesmosidic saponins, the subsequent cleavage of monosaccharide units in the C-28 position occurred preferentially. The ion peak formed by removing the entire carbohydrate chain was treated as the base peak. Multi-stage MS was further carried out taking the base peak as the parent ion followed by fracture of the carbohydrate chain at the C-3 position.

MS-MS analysis was used to determine the structure of several constituents. According to the data in Table 2, it was shown that the content of Raddeanoside A increased by 20.54% after vinegar process. This result might be caused by the ring-opening reaction of epoxy ring of other components in acid environment whose contents decreased. The increase of Raddeanoside A would be beneficial to obtain strong analgesic and anti-inflammatory effects mentioned above [36]. Besides, due to the existence of isomers, for instance, the isomers of Raddeanoside R3 and Raddeanoside R6 with the same molecular weight of 897, these mixtures should be separated before MS analysis. The fluctuation in the content of these components may have a certain improvement on their anti-inflammatory effect, which still needs to be further explored. The contents of *R17, R10 and R16* in the vinegar-processed RAR were significantly increased, likely contributing to better control of the inflammation. To the *Raddeanoside 17, R10 and R16*, the substituent groups located in R2, R3 and R4 of them are CH_3_, −gic6-gic4-rha and CH_3_, respectively. R1 represented different types of substituents attached to various types of arabinose. Thus, a better anti-inflammatory effect might be obtained using saponins with the substituents listed above. In addition to that, the anti-inflammatory effect would improve further as the content of the substituents is increased.

**Table 2:** RAR and vinegar-processed RAR retention time and peak area data statistics

Number	t_R_/min	Peak area		Peak area Drop Ratio (%)	Ingredient
		RAR	Vinegar-processed RAR		
1	15.276	9,437,539	8,163,955	13.49%↑	*Raddeanoside R9、R18、Hederacholichiside F*
2	15.788	825,649	362,244	56.13%↑	*Raddeanoside R9、R18、Hederacholichiside F*
3	16.267	1,850,801	1,762,048	4.80%↑	*Raddeanoside R9、R18、Hederacholichiside F*
4	16.836	1,859,180	3,326,004	78.90%↓	*Leonloside D*
5	19.494	2,144,743	323,379	84.92%↑	*Raddeanoside R9、R18、Hederacholichiside F*
6	19.866	350,688	2,567,728	632.20%↓	*Raddeanoside R17、R10*
7	20.287	808,195	5,424,996	571.25%↓	*Raddeanoside R16*
8	21.475	1,408,982	1,408,408	0.04%↑	*Raddeanoside R8*
9	23.143	209,933	184,567	12.08%↑	*Raddeanoside R14*
10	24.48	1,283,412	769,214	40.06%↑	*Raddeanoside R15*
11	25.19	4,043,481	3,375,726	16.51%↑	*Raddeanoside D、Hederasaponin B*
12	27.93	428,872	156,277	63.56%↑	*Raddeanoside R13*
13	33.048	639,712	159,286	75.10%↑	*Raddeanoside R13*
14	34.658	310,368	374,104	20.54%↓	*Raddeanoside A、Raddeanoside R6*
15	34.823	1,065,316	486,205	54.36%↑	*Saponin P E、Raddeanoside R12*
16	35.797	716,446	503,919	29.66%↑	*Eleutheroside K、Raddeanoside B*

Vinegar may react with the substituents on different saponins to transform R2, R3 and R4 to -CH3, −gic6-gic4-rha and CH3, respectively. Thus, the content of the anti-inflammatory active ingredient is increased accordingly. Another possible reason is that the combination of organic acid in vinegar with the alkaline substances in RAR in vinegar processing reduced the toxicity, improving the anti-inflammatory effect of vinegar-progressed RAR. As the main effective ingredient of RAR recorded in the pharmacopoeia, the content of RDA was increased by vinegar processing, contributing to the inflammation improvement. The improvement in the inflammation of other ingredients requires further study.

## Conclusions

Vinegar-processed RAR could significantly prolong the latency of the writhing reaction in mice, reduce the times of mouse writhing as well as the severity of ear swelling and foot swelling. The secretion of IL-1β, IL-6 and TNF-α proinflammatory cytokines could be remarkably inhibited. Vinegar processing could improve the analgesic and anti-inflammatory effects of RAR and reduce its toxicity. The content of twelve saponins (e.g., *Eleutheroside K*) in RAR was decreased after vinegar processing but six other types (e.g., RDA) were increased.

## Supplementary information

**Additional file 1:** Supplementary material about pictures in the manuscript.

## Data Availability

We declared that materials described in the manuscript, including all relevant raw data, will be freely available to any scientist wishing to use them for non-commercial purposes, without breaching participant confidentiality.
