# Correction to: Comparison of the acute toxicity, analgesic and anti-inflammatory activities and chemical composition changes in Rhizoma anemones Raddeanae caused by vinegar processing

**DOI:** 10.1186/s12906-020-03107-y

**Published:** 2020-10-14

**Authors:** Sha-Sha Wang, Shao-Yan Zhou, Xiao-Yan Xie, Ling Zhao, Yao Fu, Guang-Zhi Cai, Ji-Yu Gong

**Affiliations:** Changchun University of Chinese Medicine, Boshuo Road No. 1035, Jingyue High-tech Industrial Development Zone, Changchun, 130117 China

**Correction to: BMC Complement Med Ther 20, 7 (2020)**

**https://doi.org/10.1186/s12906-019-2785-0**

Following publication of the original article [1], the authors identified an error and corresponding author should be Ji-Yu Gong and not Sha-Sha Wang as previously published.

Sha-Sha Wang, Shao-Yan Zhou, Xiao-Yan Xie, Ling Zhao, Yao Fu, Guang-Zhi Cai and Ji-Yu Gong*
