# Supplementary material for: Comparison of the acute toxicity, analgesic and anti-inflammatory activities and chemical composition changes in Rhizoma anemones Raddeanae caused by vinegar processing
Source: BMC Complement Med Ther. 2020 Jan 15;20:7. doi: 10.1186/s12906-019-2785-0 (PMC7076870; doi:10.1186/s12906-019-2785-0)
Supplement: Supplementary file 1 — Additional file 1: Supplementary material about pictures in the manuscript. [file 12906_2019_2785_MOESM1_ESM.docx]

Figure B support material

Effect of RAR and its processed products on writhing reaction in mice($\overline{\text{x}}$ *± s*，*n* = 10)

| Group | Dose (mg/kg) | Latency (min) | The times of torture (15 min) |
| --- | --- | --- | --- |
| Blank Group | 0 | 4.3 ± 0.7 | 36.1 ± 3.7 |
| Indomethacin Group | 3 | 5.4 ± 0.4^**^ | 28.4 ± 2.9^*^ |
| RAR Group | 336 | 5.3 ± 0.9^**^ | 26.7 ± 3.4^*^ |
| Vinegar-  processed RAR Group | 336 | 5.7 ± 0.31^**^ | 22.9 ± 2.6^**^ |

Note: compared with the blank Group *P <0.05, **P <0.01

Figure C support material

Effects of RAR and processed products on ear swelling in mice ($\overline{\text{x}}$ ± *s*，*n* = 10)

| Group | Dose  (mg/kg) | Left Ear Weight  (mg) | Right Ear Weight  (mg) | Swell Degree  (%) |
| --- | --- | --- | --- | --- |
| Model Group | 0 | 63.85 ± 6.76 | 45.00 ± 9. 37 | 41.89% |
| Indomethacin Group | 100 | 57.76 ± 7.84* | 46.50 ± 2. 79 | 24.22% |
| RAR Group | 336 | 58. 81 ± 7. 15* | 46. 36 ± 7. 54 | 26. 86% |
| Vinegar-processed RAR Group | 336 | 53. 50 ± 4. 78** | 44. 50 ± 6. 38 | 20. 22% |

Note: compared with the model control Group *P <0.05, **P <0.01

Figure E support material

Inflammatory factor contents of IL-1β,IL-6 and TNF-α levels in each Group ($\overline{\text{x}}$ *± s*，*n* = 10)

| Group | Dose | IL-1β  (pg/mL) | IL-6  (pg/mL) | TNF-α  (pg/mL) |
| --- | --- | --- | --- | --- |
|  | (mg/kg) |  |  |  |
| Blank Group | — | 31.56 ± 6.14 | 95.72 ± 9.51 | 74.56 ± 5.49 |
| Model Group | — | 151.47 ± 10.75^**^ | 144.24 ± 12.58^**^ | 146.11 ± 4.72^**^ |
| Methotrexate Group | 5 | 54.54 ± 6.46^∆∆^ | 107.41 ± 11.21^∆∆^ | 92.38 ± 6.53^∆∆^ |
| RAR Group | 336 | 98.54 ± 12.54^∆∆^ | 126.77 ± 10.86^∆^ | 107.74 ± 5.43^∆∆^ |
| Vinegar-processed RAR Group | 336 | 68.22 ± 10.50^∆∆^ | 118.70 ± 11.54^∆∆^ | 101.89 ± 6.92^∆∆^ |

Note: compared with the blank Group: *P <0. 05, **P <0. 01, compared with the model Group: ^Δ^P < 0.05, ^ΔΔ^P < 0.01
